# A diagnostic prediction model for cardiovascular diseases (CVDs) in patients with psoriasis

**DOI:** 10.3389/fcvm.2025.1584305

**Published:** 2025-05-26

**Authors:** Xiao-Yang Guo, Guo-Hua Xue, Yue-Min Zou, Jia-Qi Chen, Shi Chen, Dong-Mei Zhou

**Affiliations:** ^1^ Beijing University of Chinese Medicine, Beijing, China; ^2^ Capital Medical University, Beijing, China; ^3^ Beijing Hospital of Traditional Chinese Medicine, Capital Medical University, Beijing, China

**Keywords:** nomogram, psoriasis, cardiovascular diseases, diagnostic, prediction model

## Abstract

**Objective:**

Individuals with psoriasis are related to a significantly increased risk of cardiovascular diseases (CVDs), the major cause of death among psoriasis patients. Prompt diagnosis and intervention of CVDs can effectively retard the progression of the disease. This study developed and validated the CVDs diagnostic prediction model for psoriasis patients.

**Methods:**

Medical records from psoriasis patients admitted to Beijing Hospital of Traditional Chinese Medicine between January 2009 and September 2024 were reviewed retrospectively. Patients were randomized as training and validation sets at the 7:3 ratio. We then selected variables through univariate logistic regression and least absolute shrinkage and selection operator (LASSO). The screened factors were subsequently incorporated in a multivariate logistic regression model for establishing the diagnostic nomogram. Moreover, this constructed model was validated internally and externally, and its performance was compared with a previous model.

**Results:**

In this study, altogether 2,685 psoriasis patients were included. Five variables were finally selected for nomogram construction, which were age, hypertension, diabetes, dyslipidemia, and fasting blood glucose (FBG). According to our results, this model achieved favorable discrimination ability, and the area under the curve (AUC) values after 500 bootstrap resampling was 0.9355 (95% CI, 0.9219–0.9491) and 0.9118 (95% CI, 0.8899–0.9338) for training and validation sets, separately. Besides, calibration curves closely matched predicted and real values for both sets. Further, as indicated by DCA results, this model showed a high net benefit at predicted probabilities below 79% and 80% of training and validation sets, separately. In total, 188 psoriasis patients were enrolled in this work, with NHANES publicly available data being utilized for external validation. The corrected AUC was 0.8293 (95% CI, 0.7574–0.9012), and the calibration and DCA curves demonstrated good accuracy and clinical utility. Additionally, the model showed an increased AUC compared with a previously published diagnostic model. Its net reclassification index (NRI) and discrimination improvement index (IDI) were positive, showing that our model was superior to the previous model.

**Conclusion:**

This study provides a cost-effective and practical tool that can assist physicians in identifying psoriasis patients at a higher CVDs risk. This may facilitate early disease diagnosis and intervention.

## 1 Introduction

Psoriasis represents the prevalent chronic inflammatory disorder that has a relapsing course and exhibits various clinical phenotypes. Its more frequent type is psoriasis vulgaris, although other subtypes, including pitting, erythrodermic, and pustular psoriasis, also occur. It can affect individuals of all ages and is currently incurable (1). In 2014, psoriasis was identified by the World Health Organisation (WHO) as a serious public health problem (2). It affects more than 125 million people worldwide (3). The prevalence of psoriasis varies greatly across different country regions. To be specific, it is generally higher in high-income countries, and the disease burden rises with the aging and growing population (4, 5). Its prevalence is 0.47% in China (6), which, given the country's large population, translates to a significant number of individuals affected, bringing considerable economic and health-related burdens on society.

Psoriasis refers to a systemic disease with multiple comorbidities including depression, psoriatic arthritis, metabolic disorders like obesity, diabetes, cardiovascular diseases (CVDs), and metabolic syndrome (7, 8). Among them, CVDs are a leading cause of death among psoriasis sufferers. Meanwhile, psoriasis is detected as a significant CVDs-related risk factor (9), while its severe forms are considered to independently predict the CVDs risk (10). Notably, CVDs are the major reason for mortality and disability worldwide (11).

The early diagnosis and intervention of CVDs exert an important effect on retarding disease progression and decreasing disease burden. However, the diagnosis of CVDs requires comprehensive testing, comprising procedures such as coronary angiography and brain imaging examinations, which can be difficult for primary care physicians. Furthermore, the symptoms of CVDs are exceedingly diverse, and some individuals exhibit atypical symptoms, leading to delays in the disease diagnosis and treatment. Thus, establishing the CVDs prediction model is crucial for psoriasis patients.

Numerous CVDs prediction models have been developed, however, most of them are based on the white race, including the Framingham prediction model, which is not applicable to the Chinese population because of racial discrepancy in CVDs prevalence (12). In addition, these models have been developed on the basis of a normal population and have restricted applicability in patients with psoriasis. Yue-Min Zou et al. (13) developed a CVDs prediction model for erythrodermic psoriasis patients at a hospital in Beijing based on a retrospective study; however, the applicability of this model to patients with psoriasis vulgaris and other subtypes of psoriasis remains unclear.

As a data visualization tool, the nomogram employs a quantitative risk prediction graph to help clinicians make faster and more informed decisions. Its use has proliferated in the field of medical research in recent years (14, 15). The present work discovered CVDs-related risk factors and established a nomogram-based diagnostic prediction model through a retrospective study. The results in this study can help clinicians to identify CVDs patients early, thereby improving the prognosis of patients with psoriasis.

## 2 Methods

### 2.1 Study design and patients

In the present retrospective study, we collected medical records from 2,685 patients with psoriasis admitted to the Department of Dermatology, Beijing Traditional Chinese Medicine affiliated to the Capital Medical University, between January 5, 2009, to September 1, 2024, to construct and internally validate the prediction model. When patients were hospitalized multiple times over the prescribed period, only the first record was used to avoid the occurrence of double counting and reduce the possibility of extra errors. Moreover, the data was accessed retrospectively on September 10, 2024.

**Figure 1:**
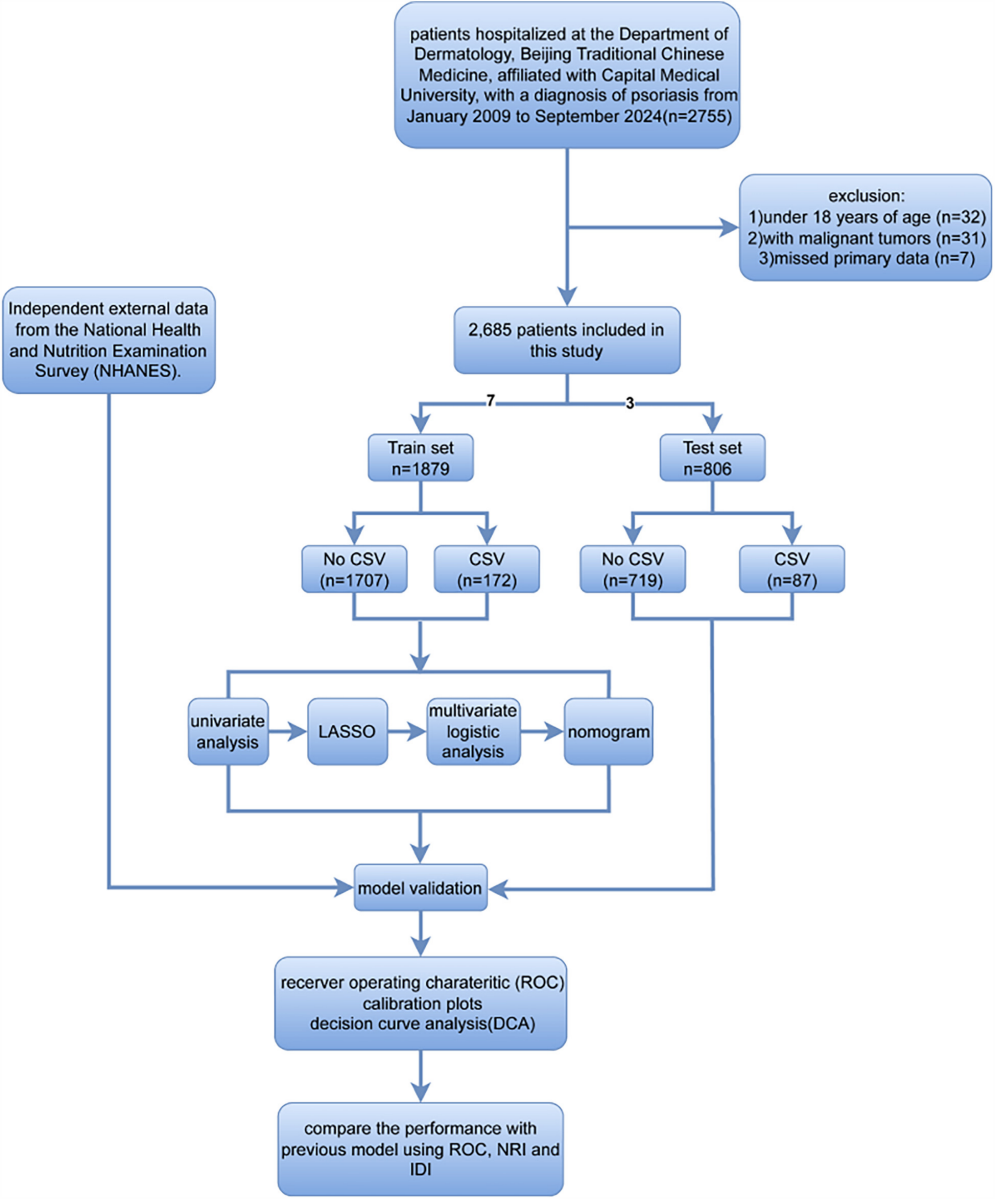
The flowchart showing patient inclusion and exclusion processes in this study.

Patients below were excluded: (1) those aged <18 years (*n* = 32), (2) those with malignant tumors (*n* = 31), and (3) missed primary data (*n* = 7). Figure 1 shows the patient selection and study procedures. Altogether there were 2,685 psoriasis patients recruited into the present work. The sample size was judged to be sufficient based on the idea of having at least ten outcome events per variable.

The externally validated statistics were collected from publicly available National Health and Nutrition Examination Surveys (NHANES) data from 2009 to 2014, which included information from a total of 188 patients with psoriasis. The NHANES is an ongoing nationwide project in the USA, which annually surveys a randomly selected cohort of approximately 5,000 representative individuals via the multistage, cluster, and stratified sampling process.

### 2.2 Ethics statement

The research has been performed in accordance with the Declaration of Helsinki. The data adopted in model establishment and internal validation were gathered from the information management platform of the hospital (the His system), and all information was anonymized, authors couldn't get any information that could identify individual participants during or after data collection. The study was approved by the Ethics Committee of Beijing Hospital of Traditional Chinese Medicine, Capital Medical University (approval number: 2023BL02-102-02), with informed consent waived due to its retrospective nature. In addition, the data employed for external validation were procured from public databases. The Ethics Review Board of the National Center for Health Statistics of the United States of America approved this survey protocol. All information collected in the survey was maintained in strict confidence. The respondents offered informed consent.

### 2.3 Diagnostic criteria

Diagnostic criteria for psoriasis were referred to the Chinese Psoriasis Diagnostic and Treatment Guidelines (2023 Edition) (16) formulated by the Chinese Medical Association, which included patients with psoriasis vulgaris (PV), pustular psoriasis (PP), erythrodermic psoriasis (EP), psoriasis arthritis (PsA). CVDs were diagnosed based on the symptoms of acute coronary syndrome, myocardial infarction, stroke and unstable angina, a prior history of coronary artery bypass graft, percutaneous coronary intervention, or the prescription of thrombolytics.

The diagnostic criteria for dyslipidemia were based on the Chinese Guideline for Lipid Management (17) (Primary Care Version 2024) issued by the Joint Committee on Chinese Lipid Management Guidelines. According to the guideline, dyslipidemia diagnosis requires meeting at least one of the following biochemical thresholds: total cholesterol (TC) ≥ 6.2 mmol/L, low-density lipoprotein cholesterol (LDL-C) ≥ 4.1 mmol/L, high-density lipoprotein cholesterol (HDL-C) < 1.0 mmol/L, or triglycerides (TG) ≥ 2.3 mmol/L. Additionally, individuals undergoing lipid-lowering therapy are likewise categorized as presenting with dyslipidemia.

Hypertension diagnosis followed the criteria established by the 2023 ESH Guidelines (18), defined as meeting any one of the following criteria (including current participation in antihypertensive therapeutic regimens):

1) Office blood pressure (OBP) ≥ 140/90 mmHg, confirmed through ≥2 separate clinic visits
2) Home blood pressure monitoring (HBPM) ≥ 135/85 mmHg averaged over 5–7 consecutive days
3) Ambulatory blood pressure monitoring (ABPM) showing: 24-hour average ≥130/80 mmHg, daytime average ≥135/85 mmHg, or nighttime average ≥120/70 mmHg.

### 2.4 Data collection

Data regarding the demographic characteristics (including age, gender, and admission time), medical history (such as a history of smoking, alcohol consumption, hypertension, diabetes, dyslipidemia, non-metabolic fatty liver disease, malignancy, and CVDs), clinical characteristics (like systolic and diastolic blood pressures), and laboratory tests (including WBC (white blood cell count), PLT (platelet count), Hb (hemoglobin content), RBC (red blood cell count), Mono (absolute monocyte count), Neut (absolute neutrophil count), Lymph (absolute lymphocyte count), NLR (neutrophil/lymphocyte ratio), SII (systemic immune inflammatory index), MCV (mean red blood cell volume), RDW-CV (red blood cell volume distribution width), Hct (hematocrit), PDW (platelet distribution width), PLT-PCT (thrombocytocrit), MPV (mean platelet volume), MCHC (mean corpuscular hemoglobin concentration), FBG (fasting blood glucose), apoE (apolipoprotein E), apoB (apolipoprotein B), apoAI (apolipoprotein AI), LPa (lipoprotein a), LDH (lactate dehydrogenase), α-HBDH (α-hydroxybutyrate dehydrogenase), CK (creatine kinase), CK-MB (creatine kinase MB), Ca (calcium), P (phosphorus), K (potassium), TP (total protein), PA (pre-albumin), ALB (albumin), GLO (globulin), GGT (γ-glutamyltransferase), TBIL (total bilirubin), DBIL (direct bilirubin), TBA (total bile acids), UA (uric acid), Crea (creatinine), Urea (blood urea nitrogen), AST (aspartate aminotransferase), ALT (alanine aminotransferase), PT (prothrombin time), FDP (fibrinogen degradation product), APTT (activated partial thromboplastin time), TT (thrombin time), D-Dimer, CRP (C-reactive protein), ESR (erythrocyte sedimentation rate), C3 (complement C3), C4 (complement C4), IgA (immunoglobulin A), IgG (immunoglobulin G), C1q (complement C1q)), MHR (monocyte-to-high-density lipoprotein ratio), and TyG (triglyceride glucose index), and other information were collected from the medical records.

Measurements of relevant indicators: (1) SII = neutrophil granulocyte count (*10^9^/L) * blood platelet count (*10^9^/L)/lymphocyte count (*10^9^/L). (2) MHR = monocyte count (*10^9^/L)/HDL (mg/dl). 5) $\mathrm{TyG}=\frac{\ln\;(FBG\times TG)}{2}$, FBG: Fasting blood glucose (mg/dl); TG: Triglycerides (mg/dl).

The externally validated data were accessible at https://www.cdc.gov/nchs/nhanes/index.htm.

### 2.5 Statistical analysis

In total, 2,685 psoriasis patients were randomized in the training and validation sets at the 7:3 ratio. Statistical analysis was carried out with SPSS software (version 26.0) and R software (version 4.2.1). Missing data were interpolated through multiple interpolation. The normally-distributed continuous data were represented by mean ± standard deviation, then analyzed by independent samples t-tests between two groups; whereas the non-normally-distributed continuous data were indicated by median ± interquartile range, then analyzed by Mann–Whitney *U*-test. Moreover, categorical data were denoted as frequencies or percentages and then analyzed using the Chi-square test. Both univariate logistic regression and least absolute shrinkage and selection operator (LASSO) algorithm were adopted for selecting variables. To be specific, variables with *P* < 0.1 in univariate logistic regression were initially identified and later subjected to the LASSO algorithm analysis through ten-fold cross-validation to prevent model overfitting. Those screened variables were subsequently adopted for multivariate logistic regression, and a backward elimination (Wald) method was employed to identify the most significant influencers for the nomogram construction.

Subsequently, internal validation was conducted using both training and internal validation sets, whereas external validation was performed on a separate external set. The area under the receiver operating characteristic (ROC) curve (AUC) was calculated for assessing model discrimination performance. Furthermore, we employed calibration curves for monitoring the concordance of the actual outcomes with the model predicted values. Clinical decision curve analysis (DCA) was carried out for assessing the clinical significance of the model. In addition, to prevent over-optimistic estimation of the model, 500 bootstrap resamples were applied to the AUC value, while 200 bootstrap resamples were applied to the DCA curves. Ultimately, the test efficacy of this model was compared to the previously published prediction models, which was accomplished by comparing the ROC and calculating the continuous net reclassification index (NRI) as well as the integrated discrimination improvement index (IDI).

## 3 Results

### 3.1 Demographic characteristics

The final cohort included 2,685 psoriasis patients, with PV being the predominant subtype (*n* = 2,028; 75.5%), followed by PsA (*n* = 293; 10.9%), EP (*n* = 236; 8.8%), and PP (*n* = 78; 2.9%). Special presentations included 47 cases (1.8%) with concomitant presentation of two subtypes, and 3 cases (0.1%) exhibiting concurrent EP, PP, and PsA. And their median age was 54 years [39; 66]. Of them, 1,800 were male (67.04%), and 259 patients developed CVDs. The participants were randomized as training (*n* = 1,879) or validation set (*n* = 806) at the 7:3 ratio. Demographic or clinical data were not significantly different in both groups (*P* ≥ 0.05). In the training set, various variables were significantly different between case and control groups, including Gender, Age, Smoking, Drinking, SBP, Hypertension, Diabetes, Dyslipidemia, PLT, NLR, FBG, GA, P, ALB, GGT, Crea, Urea, APTT, Neut, PLT- PCT, LDH, FDP, D-Dimer, ESR, C1q, Lymph, RDW-CV, PDW, IgA, WBC, α-HBDH, apoE, DBIL, Mono, C4, TP, UA, MHR, and TyG(*P* < 0.05), as displayed in S1 Table.

### 3.2 Feature selection

**Figure 2:**
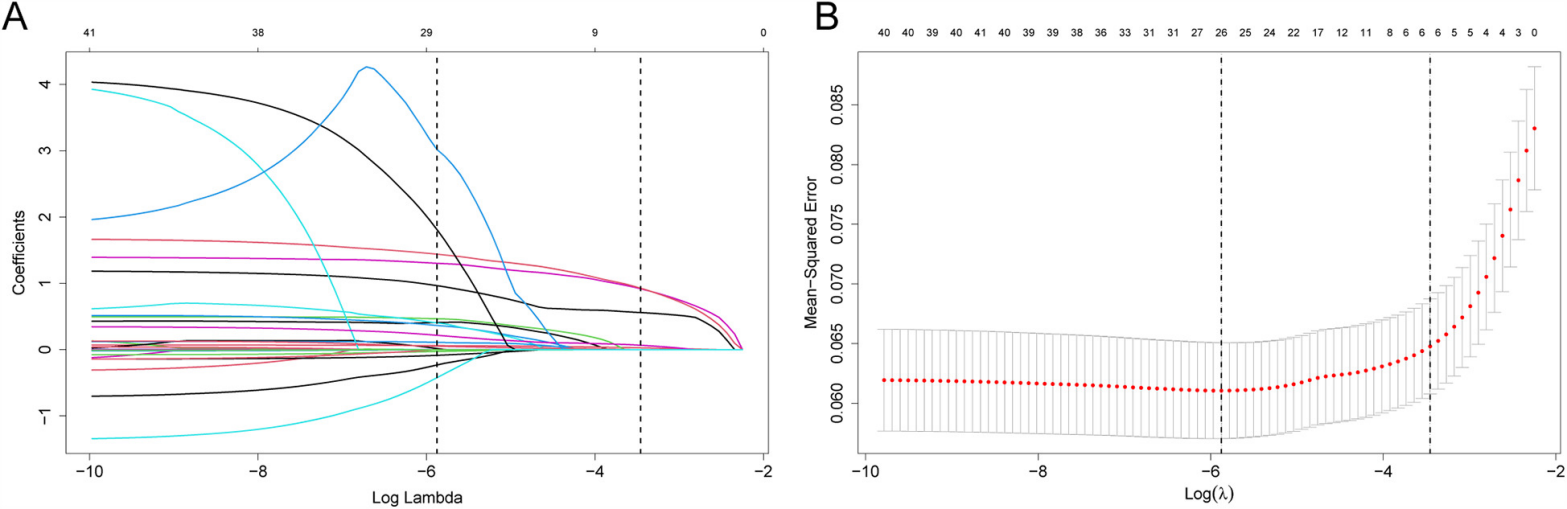
Feature screening with LASSO binary logistic regression model. **(A)** Log (Lambda) values for 39 features of LASSO model. **(B)** Optimal penalization coefficient (l) of Lasso model determined using 10-fold cross-validation and minimal criteria of training set. Left and right vertical lines stand for minimum error and cross-validated error in one standard error of the minimum, separately.

Upon univariate logistic regression, 41 variables from the validation set were significantly related to CVDs (*P* < 0.1), as shown in Table 1. Among the several potential influencing factors, variables were selected using the LASSO algorithm through 10-fold cross-validation with the choice of (1se) for five non-zero coefficients of the final model, including dyslipidemia, diabetes, hypertension, age, and FBG (Figure 2).

**Table 1:** Univariate and multivariate logistic regression for selecting CVDs-related risk factors.

Parameters	Univariate regression			Multivariate regression		
	OR	95% CI	*P*-value	OR	95% CI	*P*-value
Dyslipidemia	9.835	7.023–13.81	<0.001	4.212	2.820–6.294	<0.001
Diabetes	9.446	6.723–13.29	<0.001	1.888	1.190–2.973	0.006
Hypertension	10.99	7.817–15.64	<0.001	3.868	2.606–5.776	<0.001
Age	1.087	1.073–1.103	<0.001	1.07	1.053–1.088	<0.001
FBG	1.351	1.275–1.434	<0.001	1.171	1.086–1.263	<0.001
APTT	0.896	0.848–0.944	<0.001			
Urea	1.415	1.302–1.543	<0.001			
ALB	0.943	0.913–0.975	<0.001			
P	0.11	0.048–0.248	<0.001			
GA	1.118	1.081–1.155	<0.001			
RDW-CV	1.238	1.104–1.381	<0.001			
NLR	1.122	1.062–1.189	<0.001			
PLT	0.995	0.993–0.998	<0.001			
Drinking	1.83	1.322––2.556	<0.001			
Smoking	2.31	1.679–3.201	<0.001			
Gender	2.127	1.46–3.182	<0.001			
C1q	0.992	0.987–0.997	0.001			
LDH	1.003	1.001–1.005	0.001			
PLT-PCT	0.011	0.001–0.164	0.001			
Neut	1.105	1.041–1.17	0.001			
SBP	1.003	1.001–1.005	0.003			
Lymph	0.669	0.505–0.878	0.005			
SII	1	1–1	0.006			
WBC	1.073	1.015–1.131	0.01			
α-HBDH	1.005	1.002–1.009	0.014			
Mono	2.3	1.157–4.417	0.014			
CK	1.003	1.001–1.005	0.017			
UA	1.002	1–1.003	0.02			
LPa	1.001	1–1.001	0.025			
GGT	1.003	1–1.006	0.026			
ESR	1.009	1–1.017	0.03			
IgA	1.135	1.007–1.273	0.032			
CK-MB	1.007	1.001–1.016	0.036			
DBIL	1.075	1–1.148	0.04			
Crea	1.004	1.001–1.008	0.043			
D-Dimer	1	1–1.001	0.07			
C4	5.824	0.767–40.52	0.081			
AST	1.005	0.999–1.011	0.084			
TP	0.976	0.949–1.004	0.09			
TyG	6.31	3.828–10.43	<0.001			
MHR	16.225	2.16–117.8	0.006			

### 3.3 Nomogram prediction model construction and validation

**Figure 3:**
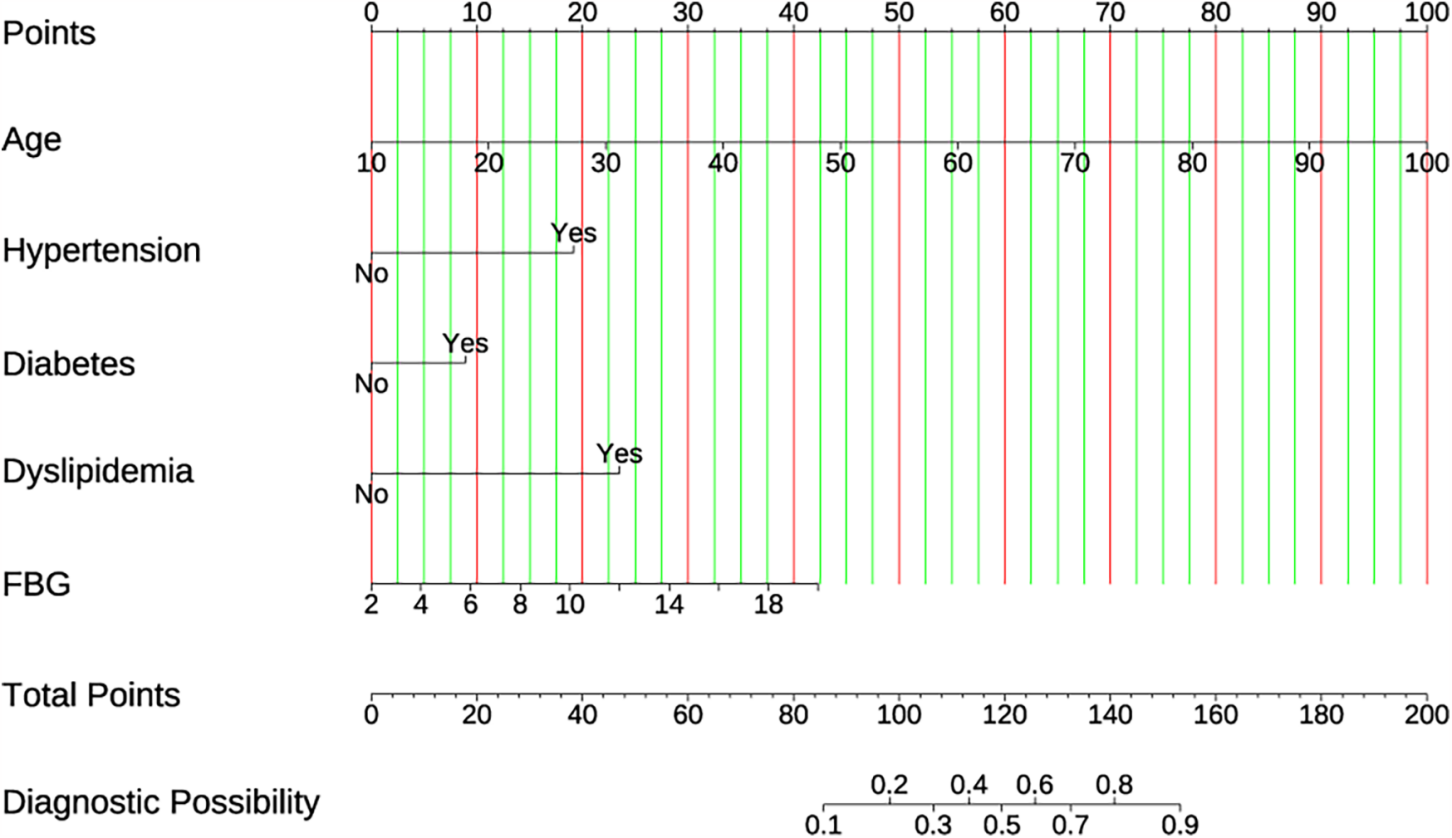
The developed CVD nomogram. The CVD nomogram for the cohort was constructed using age, hypertension, diabetes, dyslipidemia, and FBG.

To develop the prediction model, those five selected prediction variables listed above were adopted in multiple logistic regression. The results suggested that Dyslipidemia (OR: 4.213, 95% CI: 2.820–6.294, *P* < 0.001), Diabetes (OR: 1.888, 95% CI:1.190–2.973, *P* = 0.006), Hypertension (OR: 3.868, 95% CI: 2.606–5.776, *P* < 0.001), Age (OR: 1.070, 95% CI: 1.053–1.088, *P* < 0.001), and FBG (OR: 1.171, 95% CI: 1.086–1.263, *P* < 0.001) independently predicted the CVDs risk (Table 1). To improve the visibility and enable the clinical application, a nomogram was constructed utilizing the aforementioned predictors (Figure 3). The score assigned to each variable was indicative of the number of points awarded on the upper scoring axis, besides, the total score represented CVDs occurrence probability on the lower axis. The nomoscore threshold was set at 86, where scores below this value indicated low risk while those ≥86 signified high risk. At this cutoff, the model achieved 89% sensitivity and 80.7% specificity, with PPV of 31.7% and NPV of 98.6%. Furthermore, the final logistic regression coefficients presented in Table 2 allow direct score calculation without requiring the nomogram graphic.

**Table 2:** The coefficients for the final model and the full logistic equation.

Parameters	Coefficients	Logistic equation
Intercept	−8.61460	$logit\;(p)=-8.61+0.07X_{1}+1.35X_{2}+0.64X_{3}+1.44X_{4}+0.16X_{5},p=\frac{1}{1+e^{-logit(p)}}$
Age (*X*_1_, years)	0.06777	
Hypertension (*X*_2_, 1 = yes, 0 = no)	1.35276	
Diabetes (*X*_3_, 1 = yes, 0 = no)	0.63559	
Dyslipidemia (*X*_4_, 1 = yes, 0 = no)	1.43813	
FBG (*X*_5_, mmol/L)	0.15846	

*p* represents the probability of a positive event.

**Figure 4:**
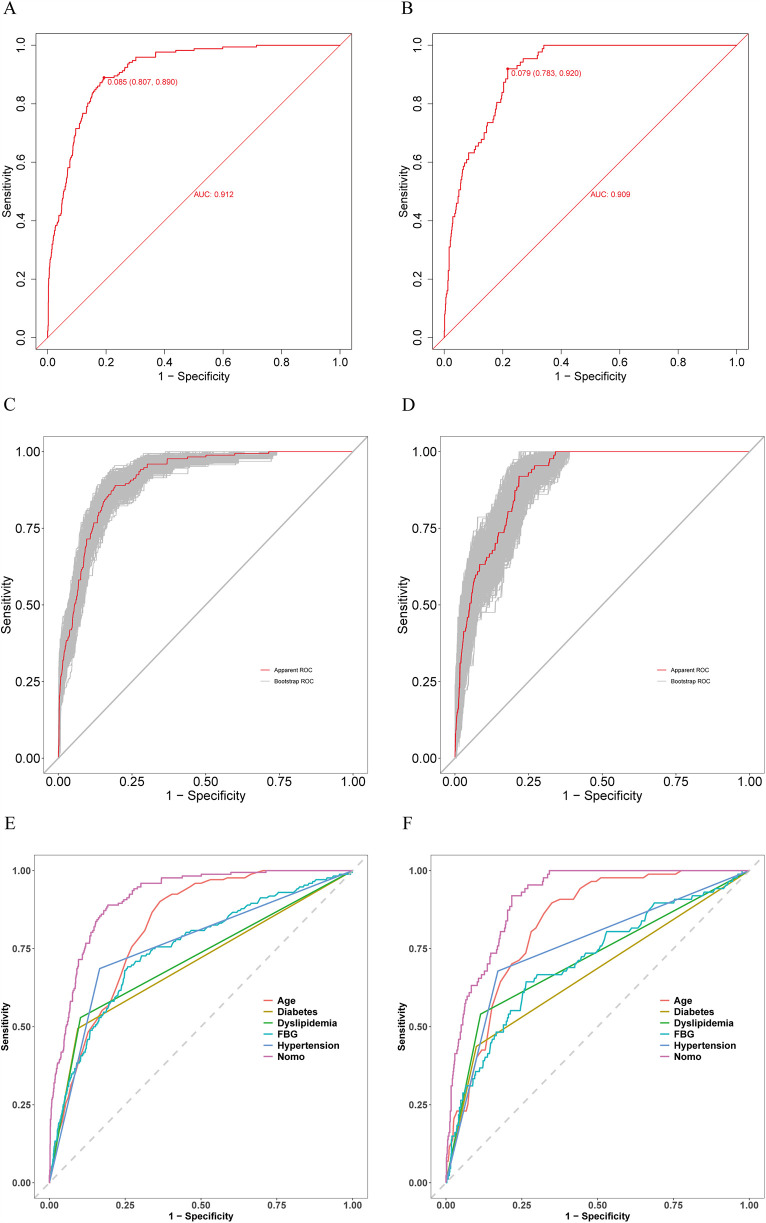
Receiver operating characteristic (ROC) curves for training and validation sets. **(A)** ROC curve for training set. **(B)** ROC curve for validation set. **(C)** ROC curve for training set after 500 bootstrap resampling. **(D)** ROC curve for validation set after 500 bootstrap resampling. **(E)** Comparison of the patient discrimination performance with prediction model by the ROC curve for training set. **(F)** Comparison of the patient discrimination performance with prediction model by the ROC curve for validation set.

The prediction model accuracy was evaluated using ROC and calibration curves, while the clinical utility was assessed through DCA curves. AUC values were 0.912 (95% CI, 0.893–0.9302; sensitivity, 89.0%; specificity, 80.7%) and 0.909 (95% CI, 0.8854–0.9328; sensitivity, 92.0%; specificity, 78.3%) for training and validation sets separately, suggesting the favorable model discrimination performance (Figures 4A,B). To reduce the optimism of the model, the 500 bootstrap resampling approach was applied in internal validation, and the AUCs for training and validation sets after internal validation were 0.9355 (95% CI, 0.9219–0.9491) and 0.9118 (95% CI, 0.8899–0.9338) separately (Figures 4C,D). Moreover, the test performance of the nomogram was compared with each predictor using the ROC curves (Figures 4E,F), demonstrating the superior discrimination ability of the nomogram.

**Figure 5:**
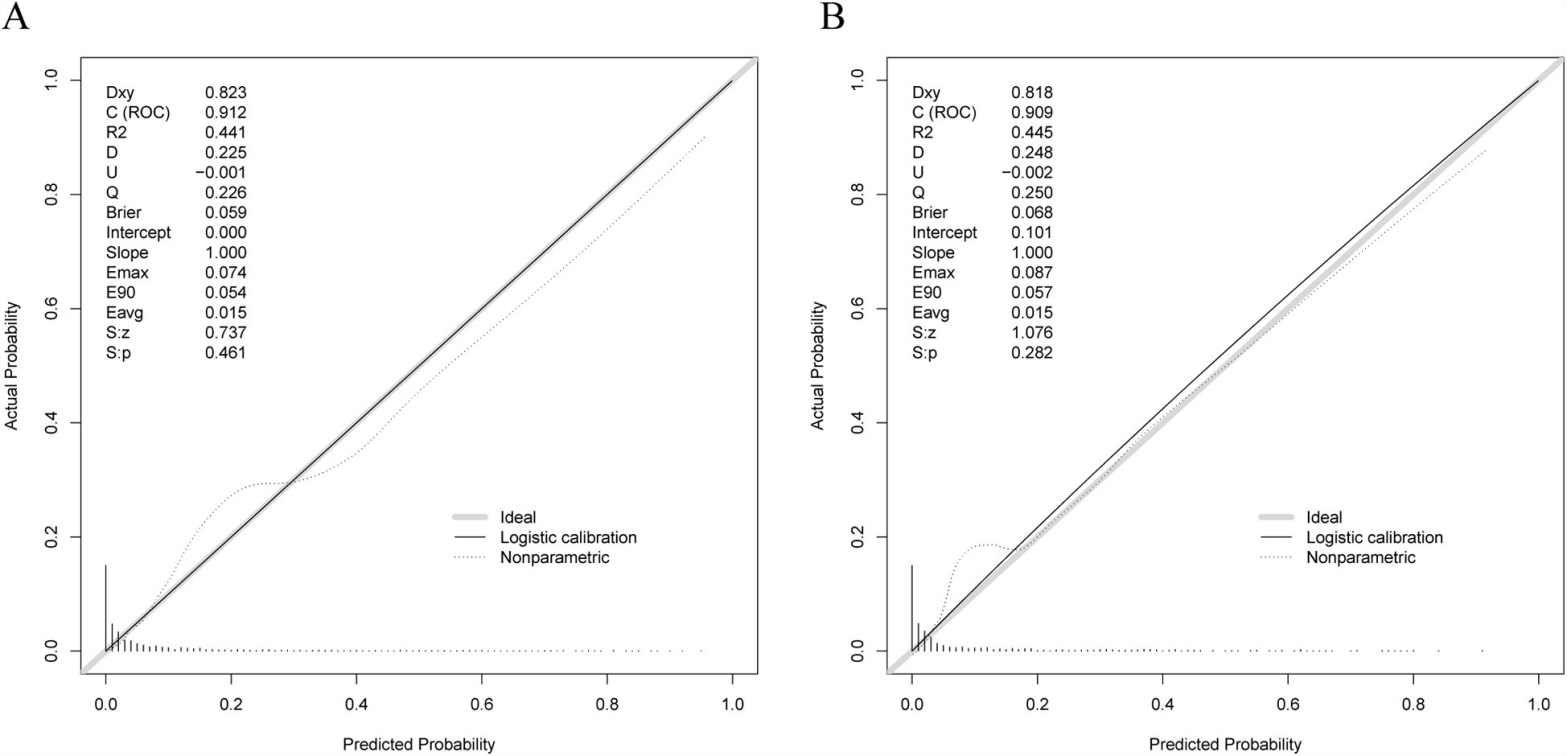
Calibration curves for training and validation sets. **(A)** Calibration curve for training set. **(B)** Calibration curve for validation set.

The model calibration curves can be observed in Figures 5A,B. As observed, the Brier value for the training set was 0.059, and *P* = 0.461 > 0.05 was obtained from the Spiegelhalter *Z*-test, while the Brier value for the validation set was 0.068, and *P* = 0.282 > 0.05 was acquired upon the Spiegelhalter *Z*-test, suggesting that the model fitted well and that the predicted and actual probabilities were quite similar.

**Figure 6:**
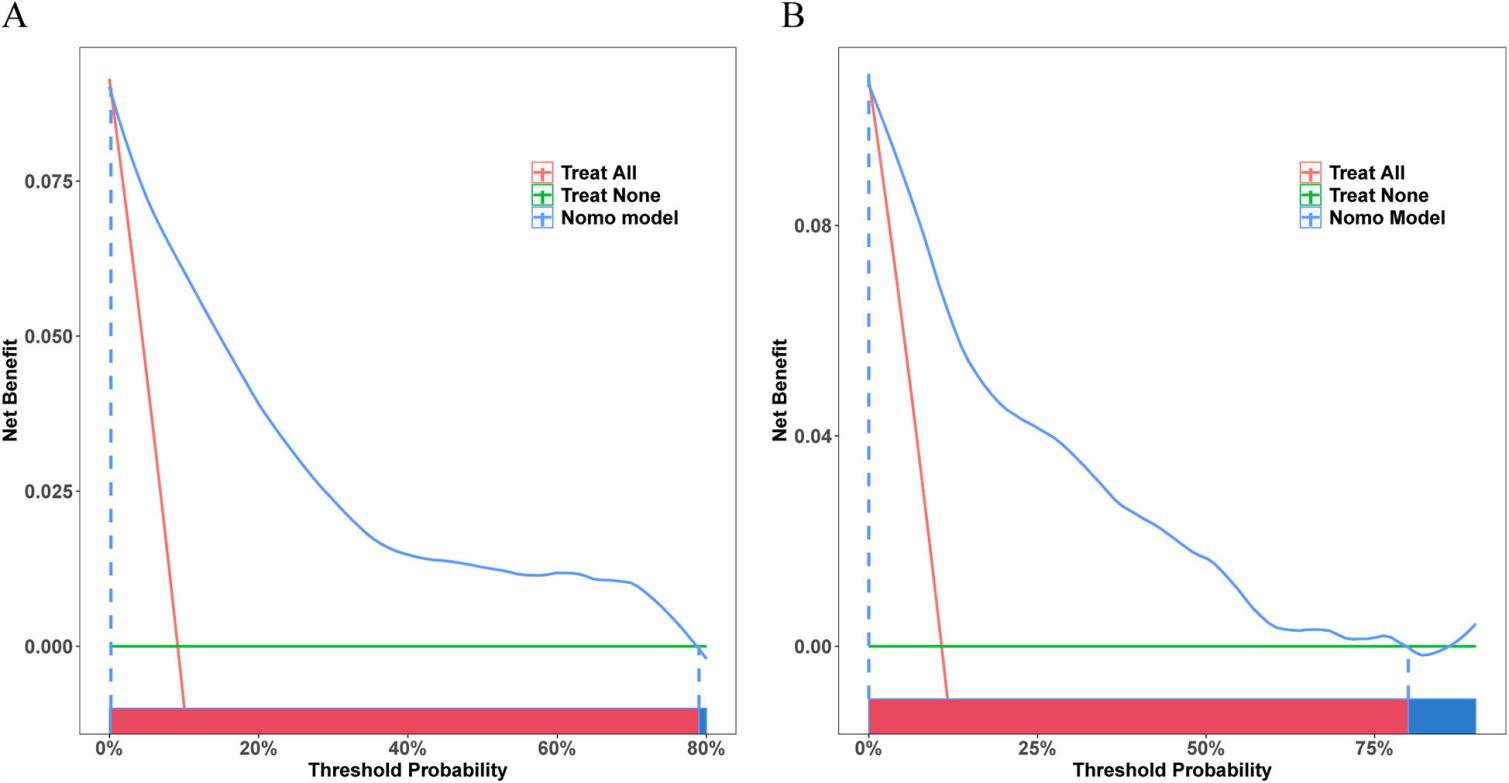
Decision curve analysis (DCA) curves for the training and validation sets. **(A)** DCA curve for the training set. **(B)** DCA curve for the validation set.

As indicated by the DCA curves, this model provided a greater net benefit if prediction probability thresholds were <79% and <80% of training and validation sets (Figures 6A,B). This implied that intervention decisions based on prediction models offered a greater benefit to patients than the alternative scenarios of treating-all-patients (diagonal line) and not-treating-all-patients (horizontal line).

### 3.4 External validation

**Figure 7:**
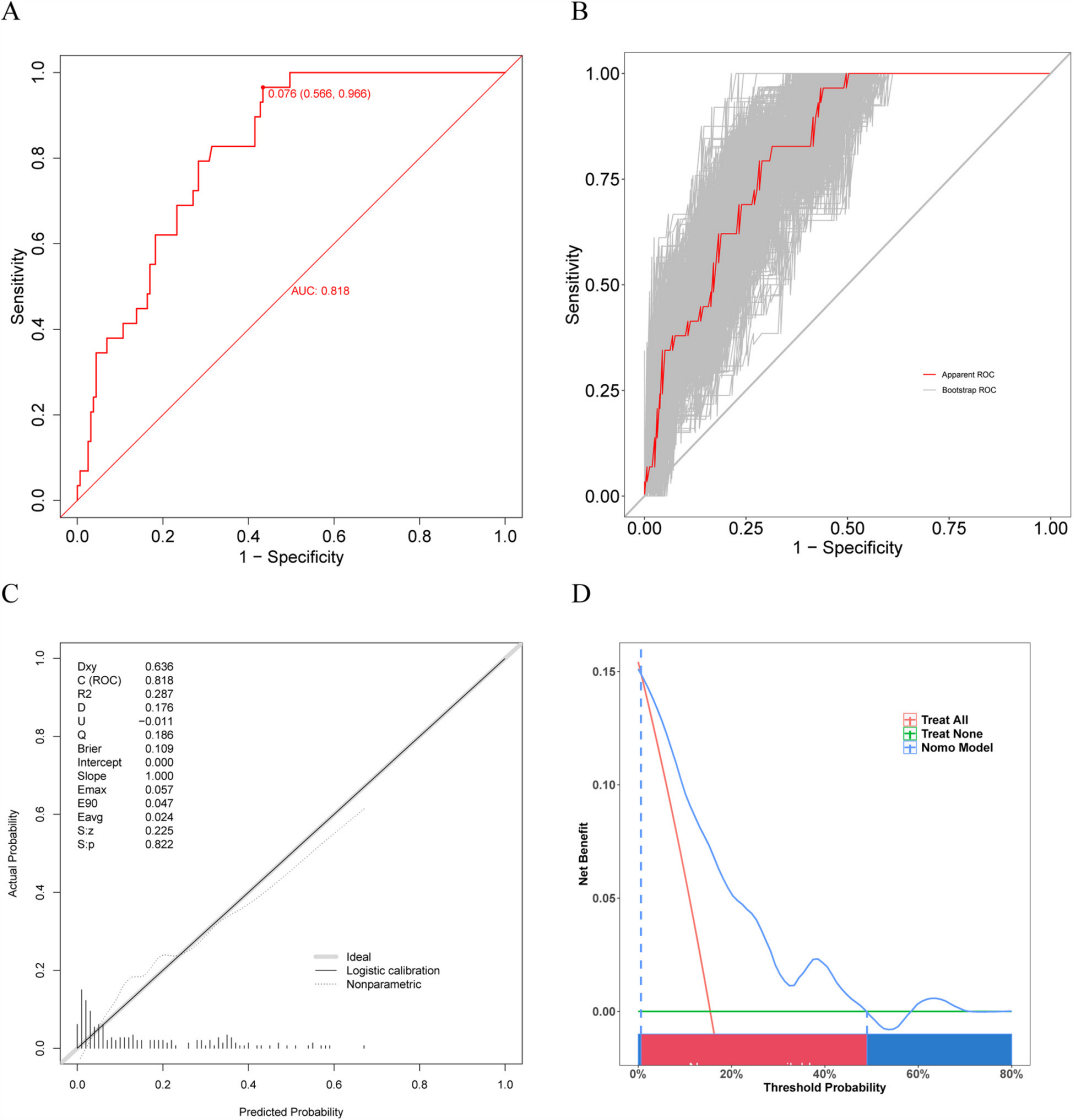
Receiver operating characteristic (ROC) curve, calibration curve, and DCA curve for the external validation set. **(A)** ROC curve for external validation set. **(B)** ROC curve for external validation set after 500 bootstrap resampling. **(C)** The calibration curve for external validation set. **(D)** The DCA curve for external validation set.

Our prediction model also showed outstanding discrimination ability for the external validation set, with the AUC value being 0.818 (95% CI, 0.7502–0.8861; sensitivity, 96.6%; specificity, 56.6%) (Figure 7A); besides, the AUC value after 500 bootstrap resampling was 0.8293 (95% CI, 0.7574–0.9012) (Figure 7B). The calibration curve is displayed in Figure 7C, with a Brier value of 0.109 and *P* = 0.822 > 0.05 from the Spiegelhalter *Z*-test, suggesting that the model was adequately calibrated. The DCA curves for the external validation set demonstrated (Figure 7D) that our model enhanced the net patient benefit if the risk threshold probability ranged from 0.6% to 49%. In conclusion, the prediction model demonstrates satisfactory prediction ability and clinical utility when applied to the external data, and thus merits further utilization.

### 3.5 Comparison with the previous diagnostic model

**Figure 8:**
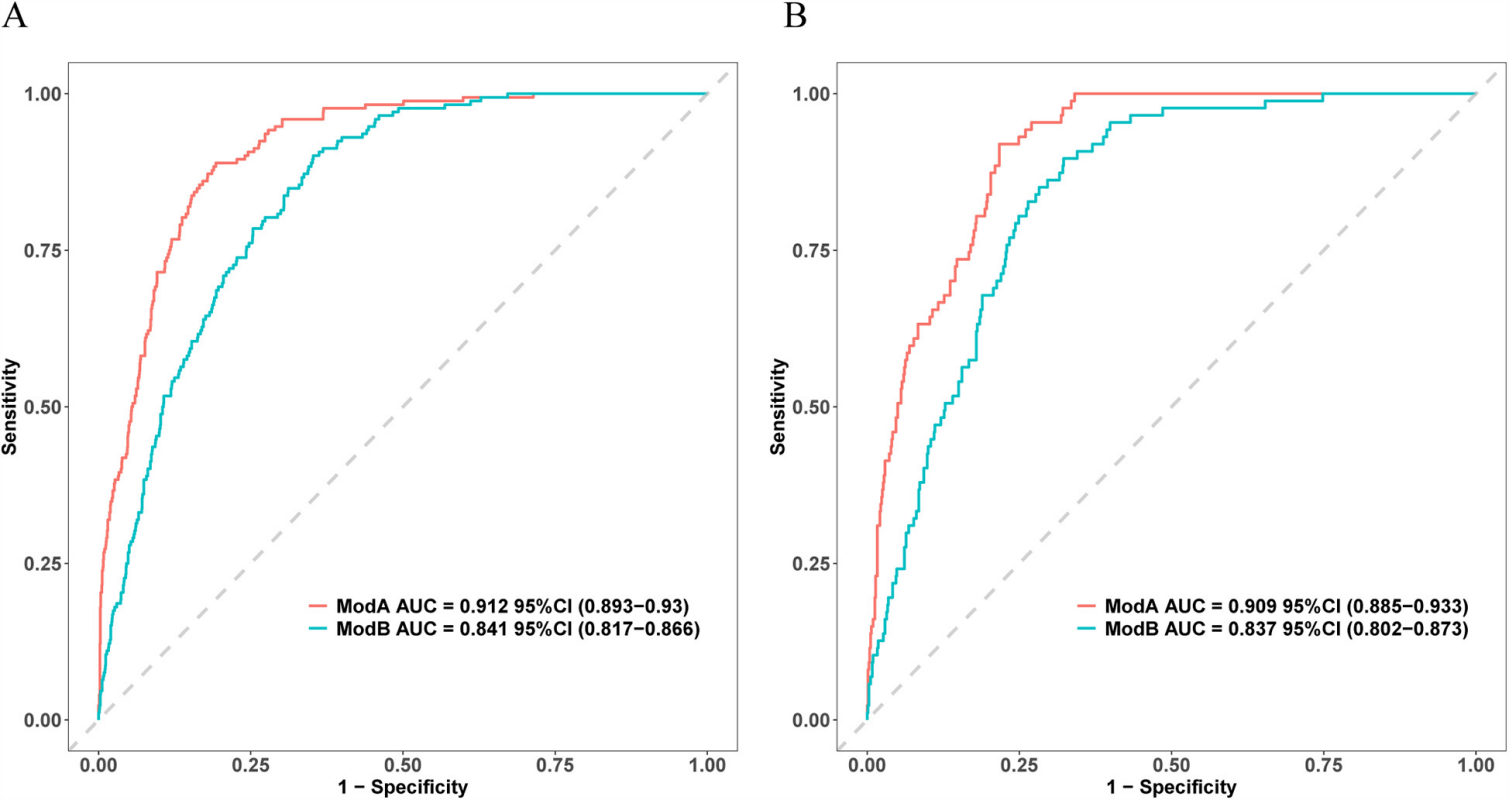
ROC curves for the present and previous prediction models. **(A)** The ROC curves for training set. **(B)** The ROC curves for internal validation set. Models A and B stand for the present and previous models, separately.

The present model and the previous prediction model were compared, which revealed that our model exhibited a superior discrimination ability in predicting CVDs (Figures 8A,B). In addition, we evaluated the performances of different prediction models in both training and validation sets with NRI and IDI. As a result, our model outperformed the previously published model in the training set, and NRI and IDI were 0.2412 (95% CI = 0.1685–0.3139, *P* < 0.001) and 0.1613 (95% CI = 0.1268–0.1959, *P* < 0.001) separately. In the validation set, NRI and IDI were 0.24 (95% CI = 0.1289–0.351, *P* < 0.001) and 0.1521 (95% CI = 0.1027–0.2014, *P* < 0.001), indicating the superior test performance of our constructed model.

## 4 Discussion

In this study, five easy-to-assess variables, including age, hypertension, diabetes, dyslipidemia, and FBG, were screened by analyzing the clinical data of patients admitted to the Department of Dermatology of the Beijing Hospital of Traditional Chinese Medicine. Subsequently, these variables were employed to create and validate a diagnostic nomogram for predicting CVD risk in psoriasis patients. According to our results, this nomogram exhibited favorable discrimination and calibration performances in internal and external validation. Moreover, as demonstrated by DCA curves, nomogram-based intervention decisions were evidently advantageous when the individual risk threshold probability fell within a specific range. Generally, the nomogram is a tool that assists physicians in classifying patients with psoriasis according to their risk profiles, enabling the early identification and diagnosis of CVDs, and providing guidance for clinical decision-making. Our model still showed good discrimination performance in a dataset based on the US demographic data as the external validation set, which may indicate that this model shows a wide range of applications and can be effectively extrapolated to a wider population.

Another crucial aspect of a prediction model lies in its ease of use. An effective prediction model should seek a balance of usability, prediction strength, and clinical applicability. The nomogram constructed in this study included five easily assessable factors frequently found in primary care settings. It reduces unnecessary measurements and has the potential to minimize the burden of care and conserve valuable health resources.

Screening rates for cardiovascular risk among patients with psoriasis are suboptimal (19). One of the main barriers to CVD prediction in psoriasis is the lack of awareness of primary care physicians (20). In addition, a recent survey showed that when assessing which implementation strategies would be most likely to improve CVD risk screening and management among patients with psoriatic disease, the highest ranked strategies among dermatologists involved clinical decision support and education (21). These findings underscore the need to develop clinically practical risk prediction models with streamlined implementation.

Age, hypertension, diabetes, and dyslipidemia are the conventional CVDs-related risk factors identified by the medical establishment. In the present work, the CVDs risk of psoriasis patients increased with increasing age, conforming to Lopez-Estebaranz et al.'s results (22) that an older age might increase the likelihood of comorbidities. Previous studies have discovered that the CVDs risk in patients with diabetes and/or hypertension increases by 1.7 times relative to subjects without these conditions (23). Dyslipidaemia significantly predicts CVDs risk, and assessment of lipid levels can help evaluate the cardiovascular risk of an individual (24). These findings are in alignment with our initial conclusions.

The FBG level has been detected as an independent predictor of CVDs in earlier research (25). It is closely linked to the burden of CVDs, particularly in China (26). Moreover, non-diabetic causes of hyperglycemia also increase the risk of CVDs (27). The higher level of FBG is associated with higher stroke and ischemic heart disease (IHD) risk (28). Even an elevated blood glucose level in the normal range may increase the risk of CVDs (25). According to our results, FBG level was closely correlated with the risk of CVDs.

Individuals diagnosed with psoriasis exhibit an elevated prevalence of hypertension, diabetes, and dyslipidemia compared with the general population (29–31), and the underlying pathogenesis is related to the long-term chronic inflammation of psoriasis. Notably, the relationship between psoriasis and CVDs is affected by the complex interplay between traditional CVDs risk factors, like diabetes, hypertension, and dyslipidemia. It is a fallacy to assume that inflammation in psoriasis is restricted to the skin. Actually, the existing evidence indicates that inflammation in the skin leads to lesion formation, which in turn stimulates an inflammatory cascade that induces systemic inflammation. This, in turn, gives rise to a number of subsequent complications, including metabolic disorders, vascular endothelial dysfunction, and plaque formation. The pathophysiology of CVDs is then influenced by these factors (32).

The inflammatory process observed during psoriasis shows the typical feature of polarisation of T helper cells (Th1 and Th17) resulting from interleukin-12 (IL-12) and IL-23. Th1 cells can generate tumor necrosis factor-α (TNF-α) and interferon-γ (IFN-γ), leading to T cell recruitment into atherosclerotic plaques and vascular endothelial dysfunction, thereby promoting plaque formation. Furthermore, these cells can directly reduce plaque stability, leading to plaque rupture (33, 34). IL-17 represents the major cytokine secreted in Th17 cells (35), which exerts pro-atherosclerotic effects (36). Moreover, the elevated level of IL-17 in plaques may lead to fibrous cap thinning, thereby leading to myocardial infarction and plaque rupture. In addition, the accumulation of IL-17 can further activate oxidative stress (37), which exerts an important effect on CVDs development (38). Additionally, IL-17 exerts an important effect on the activation of neutrophils that are involved in the pathogenesis of CVDs via various mechanisms, like promoting inflammatory responses, endothelial dysfunction, foam cell formation, lipoprotein oxidation, and interaction with macrophages (39).

Additionally, our study also suggested that inflammatory markers such as WBC (OR: 1.073, 95% CI: 1.015–1.131, *P* = 0.01), Neut (OR: 1.105, 95% CI: 1.041–1.17, *P* = 0.001), NLR (OR: 1.122, 95% CI: 1.062–1.189, *P* < 0.001), and ESR (OR: 1.009, 95% CI: 1–1.017, *P* = 0.03) markedly increased in psoriasis patients developing CVDs compared with those with no CVDs. However, model construction based on the LASSO algorithm did not include relevant inflammatory indicators. Univariate analyses demonstrated that traditional cardiovascular risk factors (e.g., age, hypertension) exhibited higher odds ratios (ORs), reflecting stronger independent associations with CVD risk. In contrast, the ORs of inflammatory indicators were significant but their clinical significance was weaker and their ORs were lower. During LASSO regression variable selection, traditional risk factors were preferentially retained owing to superior predictive capacity, whereas inflammatory biomarkers were excluded as their redundant predictive contribution failed to meet the regularization threshold. In addition, traditional risk factors can drive the inflammatory response through pathophysiologic pathways (40–42), at which point traditional risk factors indirectly encompass the effects of inflammation in the statistical model, and there is a synergistic effect between the two, leading to a nonsignificant independent effect. LASSO regression inherently prioritizes parsimony when models demonstrate comparable predictive performance. Traditional cardiovascular risk factors (e.g., age, hypertension) provided sufficient explanatory power for CVD risk stratification, as evidenced by non-significant AUC improvement with inflammatory biomarker inclusion while contravening parsimony principles. Consequently, inflammatory biomarkers were systematically excluded from the final model. From an implementation perspective, routine clinical parameters are readily available in primary care settings, whereas specialized biomarkers (e.g., ESR, NLR) necessitate specialized laboratory assays that may constrain real-world applicability. Notably, the issue of whether the incorporation of disease-specific indicators enhances the diagnostic efficacy of the model has been previously addressed in relevant literature (43). However, based on the important impact of the inflammatory response to psoriasis on CVDs, we developed an exploratory inflammatory-enhanced model (Exploratory model 1) for comparative analysis, with full specifications provided in Supplementary Material S2.

Furthermore, composite metrics such as the MHR and TyG have demonstrated robust predictive utility for subclinical CVDs in psoriasis (44, 45). However, in our analytical framework, these metrics did not exhibit superior predictive performance compared to models emphasizing traditional risk factors. Notably, given the prognostic significance of MHR and TyG, we also developed an exploratory model (Exploratory model 2) to evaluate their incremental value, as detailed in Supplementary Material S3.

Chronic inflammation in patients with psoriasis can accelerate atherosclerosis, making the actual hazard of the same traditional risk factors higher than in the general population. In contrast to conventional risk algorithms [ASCVD Risk Equation, Framingham Risk Score (FRS), SCORE Chart] derived from general population data, our model achieves parameter optimization through psoriasis-specific calibration using clinical data from 2,685 psoriatic patients, resulting in enhanced predictive accuracy and better matching the biological characteristics of this population.

It is therefore recommended that the traditional risk factors for CVDs should be more closely monitored in psoriasis patients and that blood pressure, blood glucose, and blood lipids must be actively controlled to prevent the development of CVDs in this patient group. Targeted interventions (e.g., blood pressure modulation and glycemic control) on these established factors may yield greater clinical impact by simultaneously mitigating inflammation and achieving a dual therapeutic benefit.

## 5 Limitations

Nonetheless, there are some limitations in the present work. Firstly, it should be noted that the medical records selected for this study were primarily derived from the single-center recruitment strategy (at only one tertiary hospital in Beijing) focusing exclusively on inpatients may have introduced selection bias, potentially overestimating the prevalence of psoriasis and its cardiovascular comorbidities. Because tertiary hospitals tend to admit more complex and severe cases, the psoriasis in the sample was more severe, and hospitalized patients were often admitted for acute events of CVD or metabolic disorders, the rate of comorbidities was significantly higher than that in the community population. This selection bias makes it difficult to reflect the true risk of mildly ill patients, primary care, or geographically and economically diverse groups in China, limiting the external validity of the model in China. Secondly, due to the retrospective design of this study, objective assessment indicators of patients’ disease activity (PASI, BSA) could not be systematically collected in the electronic medical record system, because such indicators are not routinely documented in clinical practice, resulting in the inability to quantitatively analyze the independent contribution of psoriasis severity to cardiovascular risk. Moreover, this may have led to selection bias thereby limiting the generalizability of the model to mild patients or outpatients. To validate the accuracy of the model, future multicentre prospective trials should be conducted. Additionally, the model's constrained external validity stems from its reliance on specific diagnostic criteria. Evolving consensus guidelines may substantially diminish the model's predictive validity. Finally, it must be acknowledged that the data collection was incomplete and did not include all the potential factors that might predict CVDs.

## 6 Conclusions

The nomogram model, established using age, hypertension, diabetes, dyslipidemia, and FBG, offers a practical approach for healthcare professionals to assess CVDs risk of psoriasis patients. The nomogram displays satisfactory discrimination capacity and notable utility in clinical applications, rendering it an appropriate approach to identify and diagnose CVDs early.

## Data Availability

The original contributions presented in the study are included in the article/Supplementary Material, further inquiries can be directed to the corresponding author.
