# Supplementary material for: A diagnostic prediction model for cardiovascular diseases (CVDs) in patients with psoriasis
Source: Front Cardiovasc Med. 2025 May 26;12:1584305. doi: 10.3389/fcvm.2025.1584305 (PMC12146384; doi:10.3389/fcvm.2025.1584305)
Supplement: Supplementary file 1 [file Table1.docx]

Supplementary Material

# **S1 Table Demographic and Clinical Features in CVDs and non-CVDs Groups.**

|  | Training set (n = 1879) | | | Validation set (n = 806) | | |
| --- | --- | --- | --- | --- | --- | --- |
| Parameters | Non-CVDs (n = 1707) | CVDs (n = 172) | P-value | Non-CVDs (n = 719) | CVDs (n = 87) | P-value |
| Gender: |  |  | < 0.001 |  |  | 0.002 |
| 0 | 587 (34.39%) | 34 (19.77%) |  | 249 (34.63%) | 15 (17.24%) |  |
| 1 | 1120 (65.61%) | 138 (80.23%) |  | 470 (65.37%) | 72 (82.76%) |  |
| Age | 51.00 [37.00;64.00] | 69.00 [63.00;77.00] | < 0.001 | 51.00 [38.00;64.00] | 69.00 [63.00;74.50] | < 0.001 |
| Smoking: |  |  | < 0.001 |  |  | 0.005 |
| 0 | 1007 (58.99%) | 66 (38.37%) |  | 432 (60.08%) | 38 (43.68%) |  |
| 1 | 700 (41.01%) | 106 (61.63%) |  | 287 (39.92%) | 49 (56.32%) |  |
| Drinking: |  |  | < 0.001 |  |  | 0.066 |
| 0 | 834 (48.86%) | 59 (34.30%) |  | 344 (47.84%) | 32 (36.78%) |  |
| 1 | 873 (51.14%) | 113 (65.70%) |  | 375 (52.16%) | 55 (63.22%) |  |
| SBP | 120.00 [111.00;130.00] | 135.00 [120.00;150.00] | < 0.001 | 120.00 [110.00;130.00] | 133.00 [120.00;140.00] | <0.001 |
| HBP | 80.00 [70.00;85.00] | 80.00 [70.00;88.00] | 0.096 | 80.00 [70.00;83.00] | 80.00 [70.00;83.50] | 0.925 |
| Hypertension: |  |  | < 0.001 |  |  | < 0.001 |
| 0 | 1424 (83.42%) | 54 (31.40%) |  | 596 (82.89%) | 28 (32.18%) |  |
| 1 | 283 (16.58%) | 118 (68.60%) |  | 123 (17.11%) | 59 (67.82%) |  |
| Diabetes: |  |  | < 0.001 |  |  | < 0.001 |
| 0 | 1547 (90.63%) | 87 (50.58%) |  | 647 (89.99%) | 49 (56.32%) |  |
| 1 | 160 (9.37%) | 85 (49.42%) |  | 72 (10.01%) | 38 (43.68%) |  |
| Hyperuricemia: |  |  | 0.154 |  |  | 0.912 |
| 0 | 1585 (92.85%) | 154 (89.53%) |  | 660 (91.79%) | 79 (90.80%) |  |
| 1 | 122 (7.15%) | 18 (10.47%) |  | 59 (8.21%) | 8 (9.20%) |  |
| Dyslipidemia: |  |  | < 0.001 |  |  | < 0.001 |
| 0 | 1532 (89.75%) | 81 (47.09%) |  | 637 (88.60%) | 40 (45.98%) |  |
| 1 | 175 (10.25%) | 91 (52.91%) |  | 82 (11.40%) | 47 (54.02%) |  |
| NAFLD: |  |  | 0.507 |  |  | 0.553 |
| 0 | 1438 (84.24%) | 141 (81.98%) |  | 606 (84.28%) | 76 (87.36%) |  |
| 1 | 269 (15.76%) | 31 (18.02%) |  | 113 (15.72%) | 11 (12.64%) |  |
| WBC | 6.48 [5.39;7.79] | 6.73 [5.74;8.24] | 0.019 | 6.53 [5.42;7.89] | 6.50 [5.36;7.62] | 0.527 |
| PLT | 232.00 [196.00;275.00] | 214.50 [171.00;252.00] | 0.001 | 230.00 [194.00;272.00] | 211.00 [174.50;247.50] | 0.001 |
| Hb | 135.00 [124.00;147.00] | 134.00 [122.00;146.00] | 0.305 | 136.00 [123.00;146.00] | 133.00 [126.00;144.50] | 0.623 |
| RBC | 4.45 [4.08;4.80] | 4.37 [4.00;4.73] | 0.160 | 4.46 [4.06;4.80] | 4.34 [4.04;4.71] | 0.187 |
| Mono | 0.49 [0.38;0.62] | 0.53 [0.40;0.67] | 0.022 | 0.49 [0.38;0.62] | 0.50 [0.38;0.62] | 0.812 |
| Neut | 3.89 [3.05;4.92] | 4.35 [3.38;5.54] | 0.001 | 3.93 [3.05;4.92] | 3.90 [3.24;4.76] | 0.832 |
| Lymph | 1.74 [1.39;2.15] | 1.60 [1.27;2.01] | 0.005 | 1.75 [1.38;2.10] | 1.50 [1.19;1.86] | 0.001 |
| NLR | 2.20 [1.64;3.06] | 2.65 [2.02;3.67] | 0.001 | 2.23 [1.68;3.14] | 2.50 [1.93;3.31] | 0.010 |
| SII | 509.60 [345.04;761.97] | 607.43 [357.15;832.21] | 0.100 | 502.15 [358.27;755.91] | 517.03 [385.97;771.67] | 0.588 |
| MCV | 91.60 [88.70;94.85] | 91.70 [88.90;95.17] | 0.417 | 91.60 [88.80;94.60] | 92.70 [89.50;96.85] | 0.010 |
| RDW-CV | 13.30 [12.80;13.80] | 13.40 [12.90;14.30] | 0.008 | 13.20 [12.80;13.90] | 13.50 [13.10;14.10] | 0.002 |
| Hct | 40.80 [37.50;43.90] | 40.10 [37.10;43.85] | 0.290 | 40.90 [37.05;43.95] | 40.50 [38.15;43.30] | 0.885 |
| PDW | 16.30 [15.80;16.70] | 16.40 [16.00;16.80] | 0.017 | 16.30 [15.90;16.70] | 16.50 [16.10;16.95] | 0.007 |
| PLT-PCT | 0.20 [0.17;0.24] | 0.19 [0.16;0.22] | 0.001 | 0.20 [0.17;0.24] | 0.17 [0.15;0.21] | 0.001 |
| MPV | 8.60 [7.90;9.60] | 8.70 [7.88;9.60] | 0.432 | 8.60 [7.80;9.60] | 8.60 [7.90;9.45] | 0.647 |
| MCHC | 333.00 [328.00;338.00] | 333.00 [327.75;337.00] | 0.956 | 332.00 [327.00;337.00] | 331.00 [327.00;335.50] | 0.339 |
| FBG | 4.95 [4.57;5.57] | 6.23 [5.32;8.66] | 0.001 | 5.00 [4.59;5.70] | 6.08 [4.99;8.73] | 0.001 |
| GA | 13.00 [11.90;14.20] | 14.45 [12.57;17.65] | 0.001 | 12.90 [11.80;14.30] | 13.90 [12.30;16.15] | 0.001 |
| apoE | 37.70 [31.40;45.10] | 35.15 [30.35;42.90] | 0.021 | 38.00 [31.70;45.45] | 37.00 [29.90;43.85] | 0.298 |
| apoB | 0.89 [0.73;1.06] | 0.85 [0.69;1.02] | 0.075 | 0.88 [0.73;1.03] | 0.82 [0.67;1.03] | 0.212 |
| apoAI | 1.14 [1.00;1.32] | 1.15 [0.98;1.30] | 0.817 | 1.14 [0.99;1.31] | 1.16 [1.02;1.35] | 0.282 |
| LPa | 121.10 [55.30;271.50] | 144.75 [63.17;310.52] | 0.116 | 132.60 [60.55;244.60] | 168.90 [71.70;297.55] | 0.073 |
| LDH | 160.50 [138.60;190.75] | 175.00 [146.05;204.98] | 0.001 | 160.70 [137.75;187.60] | 176.90 [149.10;216.80] | 0.001 |
| α-HBDH | 121.90 [105.50;141.00] | 127.10 [110.00;148.48] | 0.019 | 120.50 [106.65;140.05] | 127.00 [109.60;155.40] | 0.044 |
| CK | 71.00 51.00;98.60] | 77.65 52.95;115.35] | 0.055 | 72.00 53.05;98.75] | 78.00 [52.55;125.05] | 0.241 |
| CK-MB | 9.90 [6.90;12.90] | 9.55 [4.48;14.00] | 0.401 | 9.60 [6.45;13.05] | 9.90 [3.83;12.95] | 0.586 |
| Ca | 2.27 [2.20;2.34] | 2.27 [2.21;2.34] | 0.882 | 2.27 [2.20;2.35] | 2.25 [2.18;2.33] | 0.232 |
| P | 1.26 [1.13;1.40] | 1.19 [1.06;1.29] | 0.001 | 1.27 [1.15;1.40] | 1.16 [1.04;1.26] | 0.001 |
| K | 4.00 [3.80;4.20] | 4.00 [3.77;4.25] | 0.668 | 4.00 [3.81;4.19] | 3.98 [3.78;4.25] | 0.875 |
| TP | 64.90 [61.40;68.30] | 63.85 [60.50;67.40] | 0.040 | 64.80 [61.10;68.40] | 63.10 [60.35;68.05] | 0.048 |
| PA | 215.10 [170.40;256.00] | 217.85 [170.70;264.45] | 0.545 | 219.80 [174.50;263.70] | 215.10 [163.15;245.40] | 0.210 |
| ALB | 38.90 [36.20;41.45] | 37.75 [34.60;40.12] | 0.001 | 39.00 [36.30;41.50] | 38.00 [35.25;40.25] | 0.015 |
| GLO | 25.80 [23.30;28.70] | 26.00 [24.00;29.10] | 0.171 | 25.60 [23.10;28.80] | 25.80 [23.50;28.05] | 0.613 |
| GGT | 19.50 [13.80;28.60] | 24.50 [17.98;36.12] | 0.001 | 20.00 [14.20;28.05] | 24.50 [17.20;38.70] | 0.003 |
| TBIL | 11.60 [9.30;14.60] | 12.32 [9.30;14.87] | 0.417 | 11.40 [8.90;14.20] | 11.10 [8.85;15.90] | 0.532 |
| DBIL | 3.20 [2.50;4.26] | 3.40 [2.70;4.60] | 0.021 | 3.20 [2.41;4.05] | 3.70 [2.81;4.80] | 0.002 |
| TBA | 3.80 [2.30;6.90] | 4.30 [2.48;7.03] | 0.254 | 3.90 [2.30;6.40] | 4.30 [2.55;6.85] | 0.121 |
| UA | 357.40 [291.60;426.75] | 374.95 [310.28;440.30] | 0.040 | 362.20 [299.95;438.35] | 352.00 [309.10;424.50] | 0.681 |
| Crea | 66.00 [56.00;77.00] | 74.50 [59.58;90.05] | 0.001 | 66.60 [56.15;76.50] | 67.40 [58.10;76.30] | 0.544 |
| Urea | 4.10 [3.28;5.11] | 5.26 [4.13;6.86] | 0.001 | 4.24 [3.37;5.28] | 5.17 [3.94;5.98] | 0.001 |
| AST | 19.90 [16.10;25.05] | 19.55 [15.67;23.77] | 0.212 | 20.00 [16.30;25.70] | 19.40 [16.00;25.80] | 0.676 |
| ALT | 18.00 [12.50;27.05] | 17.50 [13.30;25.75] | 0.833 | 18.40 [13.15;29.50] | 17.70 [12.85;26.05] | 0.219 |
| PT | 11.10 [10.50;11.70] | 11.00 [10.50;11.60] | 0.518 | 11.00 [10.50;11.65] | 10.90 [10.50;11.55] | 0.935 |
| FDP | 1.10 [0.53;2.01] | 1.38 [0.80;2.74] | 0.001 | 1.14 [0.56;2.00] | 1.36 [0.69;2.30] | 0.172 |
| APTT | 31.90 [29.90;33.90] | 30.85 [28.80;33.02] | 0.001 | 32.20 [30.00;34.10] | 30.80 [29.25;32.95] | 0.004 |
| TT | 14.40 [13.60;15.20] | 14.30 [13.60;15.30] | 0.893 | 14.30 [13.70;15.20] | 14.40 [13.70;15.20] | 0.877 |
| D-Dimer | 0.15 [0.07;0.93] | 0.22 [0.10;88.00] | 0.001 | 0.17 [0.08;1.35] | 0.20 [0.08;0.81] | 0.738 |
| CRP | 3.24 [1.60;8.34] | 4.25 [1.79;11.80] | 0.051 | 3.00 [1.65;8.78] | 4.00 [1.78;12.65] | 0.134 |
| ESR | 7.00 [3.00;14.00] | 9.00 [5.00;18.50] | 0.001 | 7.00 [3.00;14.00] | 8.00 [4.50;15.00] | 0.239 |
| C3 | 1.06 [0.91;1.21] | 1.08 [0.95;1.22] | 0.126 | 1.06 [0.92;1.22] | 1.10 [0.92;1.23] | 0.593 |
| C4 | 0.22 [0.18;0.27] | 0.23 [0.19;0.28] | 0.030 | 0.22 [0.18;0.26] | 0.22 [0.18;0.26] | 0.683 |
| IgA | 2.38 [1.75;3.15] | 2.58 [1.95;3.51] | 0.017 | 2.28 [1.71;3.12] | 2.31 [1.83;3.52] | 0.295 |
| IgG | 10.65 [8.99;12.40] | 10.50 [8.52;12.29] | 0.411 | 10.57 [8.84;12.34] | 10.18 [8.55;12.20] | 0.472 |
| C1q | 167.52 [147.28;191.53] | 158.83 [140.28;179.93] | 0.001 | 167.31 [150.30;189.60] | 158.56 [138.38;181.71] | 0.021 |
| MHR | 0.39 [0.34;0.44] | 0.40 [0.35;0.46] | 0.008 | 0.39 [0.35;0.43] | 0.41 [0.36;0.45] | 0.077 |
| TyG | 4.63 [4.46;4.82] | 4.80 [4.60;5.03] | < 0.001 | 4.63 [4.46;4.83] | 4.75 [4.58;4.96] | < 0.001 |
